# Comparison of transcriptome alterations induced by pendimethalin or its commercial formulation Stomp Aqua in human MCF-7, MCF-10 A and MCF-12 A mammary epithelial cells

**DOI:** 10.1186/s13104-023-06327-w

**Published:** 2023-04-25

**Authors:** Robin Mesnage, Helin Omriouate, Michael N Antoniou

**Affiliations:** 1 Gene Expression and Therapy Group, Faculty of Life Sciences & Medicine, Department of Medical and Molecular Genetics, King’s College London, Guy’s Hospital, London, SE1 9RT UK; 2 Buchinger Wilhelmi Clinic, Wilhelmi-Beck-Straße 27, 88662 Überlingen, Germany

**Keywords:** Herbicides, Pendimethalin, Breast cancer, Endocrine disruptor, Transcriptome

## Abstract

**Objective:**

The toxicology of herbicides, which are currently in use is under-explored. One highly used but under investigated herbicide is pendimethalin. Here we mined high-throughput data from the US National Toxicology Program (NTP) to identify whether pendimethalin possesses an estrogenic capability in human cells. We also evaluated effects of pendimethalin and its reference commercial formulated herbicide Stomp Aqua on the transcriptome profile of three human mammary epithelial cell lines, cancerous MCF-7 and non-cancerous MCF-10 A and MCF-12 A to see whether this compound could have endocrine disrupting effects and if co-formulants present in the commercial formulation could amplify its toxicity.

**Results:**

The data mined from the US NTP database suggests that pendimethalin activates estrogen receptors at a concentration of approximately 10?M. MCF-7, MCF-10A and MCF-12A cells were exposed to 10 ?M pendimethalin and Stomp Aqua at an equivalent concentration. Transcriptome analysis showed changes in gene expression patterns implying that pendimethalin affected ubiquitin-mediated proteolysis and the function of the spliceosome. The formulated pendimethalin product Stomp Aqua gave comparable effects suggesting pendimethalin was responsible for the observed transcriptome alterations. Given the lack of information on the exposure to this pesticide, our study prompts the need for biomonitoring studies, especially under occupational use scenarios, to understand if low level exposure to pendimethalin could have endocrine disrupting effects on populations exposed to this compound. A deeper understanding of the exposure and mechanisms of action of this endocrine-disrupting pesticide is needed.

**Supplementary Information:**

The online version contains supplementary material available at 10.1186/s13104-023-06327-w.

## Introduction

Exposure to herbicides have been implicated in the development of numerous human diseases, with an increasing number of studies published every year revealing negative health effects, which were not always detected in studies conducted for regulatory purposes [1]. Effects of pesticides in human populations can remain undetected for several decades as has been the case for DDT [2]. For major herbicides such as 2,4-D, their carcinogenicity is still debated despite decades of use, and evidence of genotoxicity [3] and numerous epidemiologic studies [4, 5]. This can be due to the lack of sensitivity of toxicity tests, which do not always detect metabolic disturbances leading to chronic diseases following long-term exposure [6]. The toxicology of major herbicides, which are currently in use remains under-explored. Although a large number of studies have investigated the toxicity of glyphosate, very few studies have been undertaken to evaluate the effects of other herbicides such as pendimethalin, which is used at a large scale in the UK. In this study we mined high-throughput data from the US National Toxicology Program (NTP) to identify whether pendimethalin has an estrogenic capability in human cell line model systems. As these results suggested that pendimethalin could act as an estrogen mimic, we evaluated pendimethalin and its reference commercial formulated herbicide Stomp Aqua for their ability to disturb the biology of three human mammary epithelial cell lines (MCF-7, MCF-10 A, MCF-12 A) in order to gain insight as to whether this herbicide could have endocrine disrupting effects and if co-formulants in the commercial formulation could amplify such outcomes.

## Materials and methods

### Cell culture

Pendimethalin was purchased from Merck KGaA (Sigma Aldrich®, Gillingham, Dorset, UK). The pendimethalin formulation Stomp aqua (BasF, 455 g/L Pendimethalin) was purchased from PestControl Expert S.R.L. MCF-7 and MCF-10 A cell lines were purchased from the ECACC General Cell Collection (UK Health Security Agency, Porton Down, Salisbury, UK). MCF-12 A cells were a gift from Dr Elisabete Silva (Brunel University of London, UK). MCF-7 cells were grown in Dulbecco’s modified Eagle’s medium (DMEM; ThermoFisher Scientific, Loughborough, UK) supplemented with 2 mM glutamine (GE Healthcare Life Sciences, Chalfont St Giles, UK), 10 µg/ml penicillin/streptomycin (ThermoFisher Scientific) and 5% foetal bovine serum (FBS, Merck). MCF-10 A and MCF-12 A cells were cultured in DMEM-F12 (ThermoFisher Scientific) supplemented with 20ng/ml epidermal growth factor (EGF), 0.5 µg/ml hydrocortisone (ThermoFisher Scientific), 100ng/ml cholera toxin, 10 µg/ml insulin (ThermoFisher Scientific), 10 µg/ml penicillin/streptomycin (ThermoFisher Scientific) and 5% horse serum (ThermoFisher Scientific). All cell cultures were maintained at 37^o^C in a 5% CO_2_ air atmosphere in 75cm^2^ flasks (Corning, Tewksbury, USA).

### Culture of cells for transcriptome analysis

MCF-7, MCF-10 A and MCF-12 A cells were seeded at a density of 8 × 10^3^ cells/mL in a volume of 250 µl/well in 48-well plates (Corning, Tewksbury, USA) and exposed to either 10 µM pendimethalin or the commercial formulation Stomp Aqua at the same pendimethalin equivalent concentration for 48 h. To avoid interference from estrogen in the sera added to the media, FBS and horse serum were stripped of steroid hormones by treatment with charcoal as previously described [7]. A total of 5 cell cultures for each treatment and untreated control group were set up for transcriptome analysis.

### Transcriptome analysis

Total RNA was then extracted using the Qiagen RNeasy Mini Kit. RNA Library Preparation and cDNA Sequencing was performed under contract with GENEWIZ (Leipzig, Germany). Library preparation for RNA sequencing employed the NEBNext Ultra II RNA Library Prep Kit for Illumina following the manufacturer’s instructions (NEB, Ipswich, MA, USA). The sequencing libraries were multiplexed and loaded on the flow cell of the Illumina NovaSeq 6000 instrument according to the manufacturer’s instructions. The samples were sequenced using a 2 × 150 Pair-End (PE) configuration v1.5.

The data collected from the cDNA Sequencing were analyzed in R studio as previously described [8]. In brief, transcript abundance was against a reference transcriptome (Homo sapiens GRCh38 cDNA fasta). Differential gene expression analysis was done using DESeq2, and a KEGG pathway enrichment analysis was ultimately conducted using the goseq R Bioconductor package.

### ToxCast data

Publicly available data from the ToxCast program was analyzed using the Integrated Chemical Environment (ICE) web resource developed by the US National Toxicology Program Interagency Center for the Evaluation of Alternative Toxicological Methods [9]. We focused on the results of high-throughput assays related to endocrine processes. These include assays measuring different aspects of endocrine mode of action such as activation of transcription through receptor response elements, formation of receptor dimers or receptor activation, for glucocorticoids, estrogen, androgen and thyroid hormone signalling, as well as cholesterol transport and aromatase enzyme activity.

## Results and discussion

**Fig. 1:**
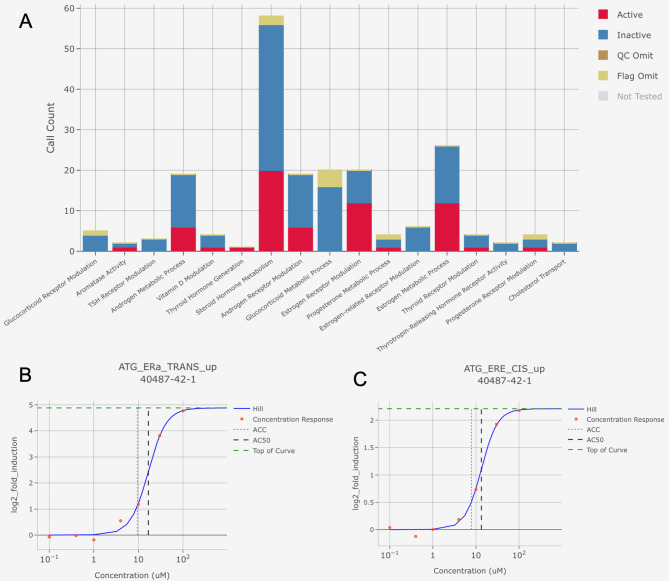
Pendimethalin is predicted to activate estrogen signalling pathways in high-throughput tissue culture cell assays. The US ToxCast database was scrutinized using the Integrated Chemical Environment (ICE) web resource. **A.** The stacked bargraph shows whether different categories of assays resulted in active or inactive results. The dose response data for Attagene trans (**B**) and cis-Factorial (**C**) assays were extracted. Pendimethalin dose response curve (0.1–100 µM) is expressed as fold induction

We first assessed if pendimethalin possessed an endocrine mode of action by mining the high-throughput assays of the ToxCast database. A total of 314 assays were found for 17 endocrine categories (Fig. 1A). Most positive scores were attributed to steroid hormone metabolism, and in particular the activation of estrogen receptor signalling pathways. Effects on the estrogen receptor pathway were mixed with 12 assays predicting that pendimethalin activated estrogen receptors, whilst 8 assays found that this compound was not estrogenic. Pendimethalin was suggested to activate estrogen receptors at a concentration of approximately 10 µM in Attagene assays ATG_ERa_TRANS_up (Fig. 1B) and ATG_ERE_CIS_ up (Fig. 1C) reporter gene assays measuring mRNA in HepG2 cells, which has been proven to be a reliable cell line for the evaluation of endocrine disrupting effect of chemicals [10].

We next sought to obtain mechanic insight into the potential endocrine disrupting effects of pendimethalin and its commercial formulation Stomp Aqua by conducting a transcriptome analysis. The hormone-dependent human breast cancer cell line MCF-7 and the human non-cancerous immortalised mammary epithelial cell lines MCF-12 A and MCF-10 A were exposed to either pendimethalin or Stomp Aqua at a concentration of 10 µM as this was found to be estrogenic in the ToxCast assays (Fig. 1). Transcriptome profiling was by RNA-seq on the Illumina sequencing platform with cells harvested and analysed following 48 h of exposure.

**Fig. 2:**
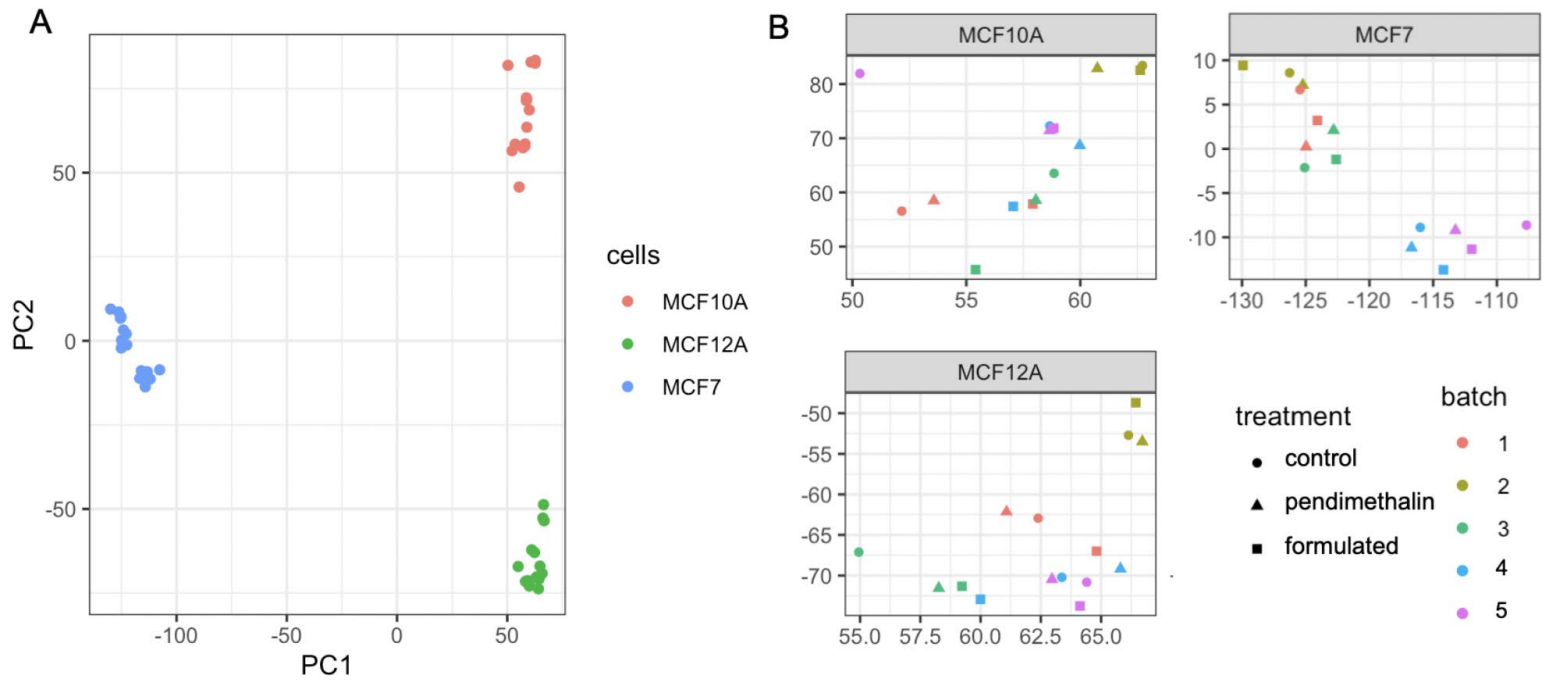
PCA reveals variations in gene expression profiles after exposure of MCF-7, MCF-10 A and MCF-12 A human mammary epithelial cell lines to pendimethalin. PCA showing the clustering of the samples along the PC1 and PC2 axes coloured by cell line **(A)** or by treatment extracted from MCF-10 A (cluster 1), MCF-12 A (cluster 2) and MCF-7 (cluster 3) **(B)**

**Fig. 3:**
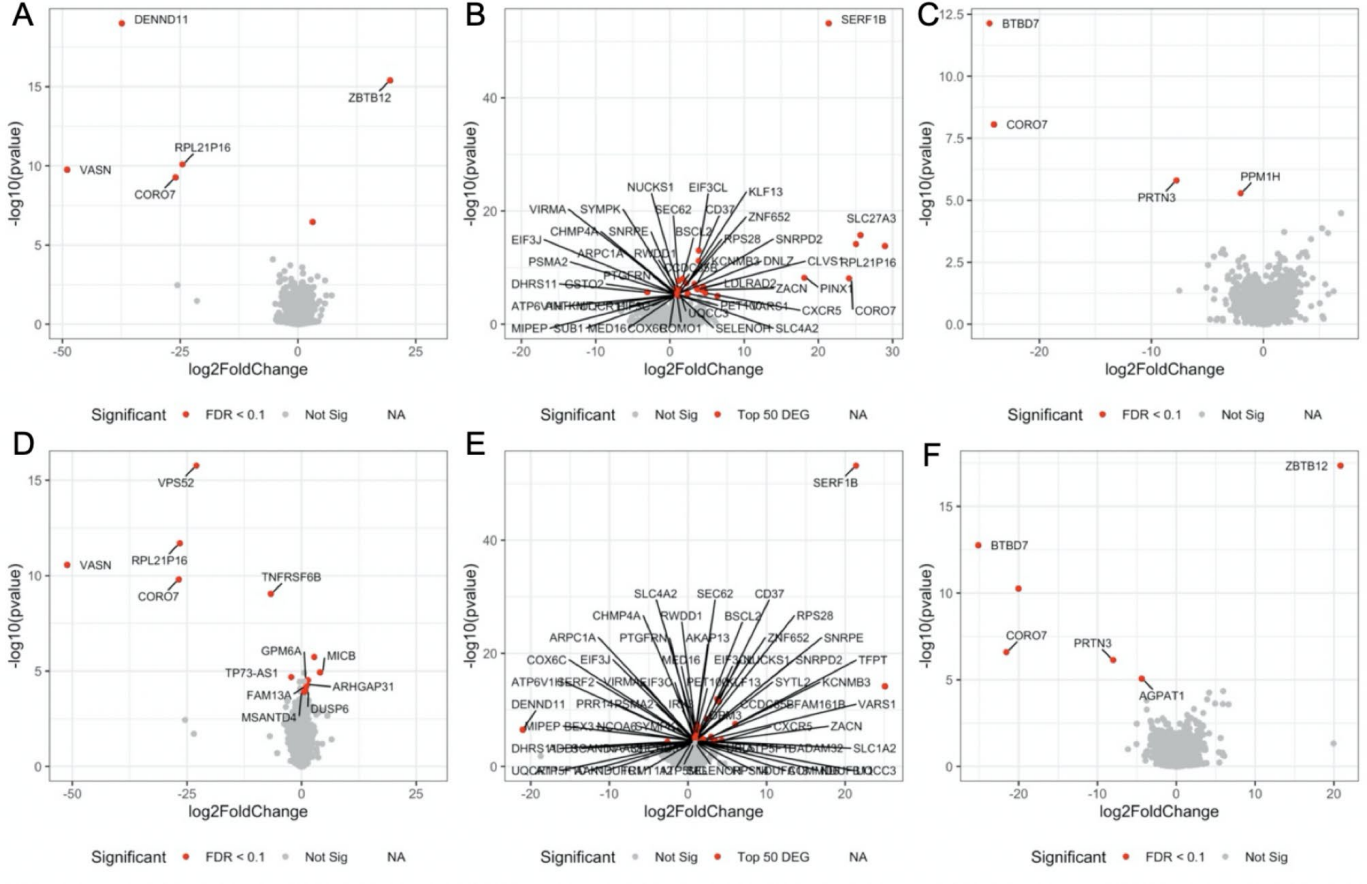
Volcano plots showing genes, which had their expression altered after exposure to pendimethalin or its formulation Stomp Aqua. Volcano plots showing fold-changes and statistical significance in the expression of genes affected by exposure to pendimethalin (**A**, in MCF7; **B**, in MCF-10 A; **C**, in MCF-12 A) or its commercial formulation Stomp Aqua (**D**, in MCF7; **E**, in MCF-10 A; **F**, in MCF-12 A). Genes which have their expression significantly altered (FDR < 0.1) in MCF-7 or MCF-12 A cells are shown in red. Given the large number of genes having their expression significantly altered (FDR < 0.1) in MCF-10 A, only the 50 genes most altered in their expression are shown

A principal component analysis (PCA) was conducted to explore the major source of variation in the transcriptome data (Fig. 2A). The first 2 components were mainly driven by the separation between the three cell types (Fig. 2A) and the treatments didn’t cluster the data (Fig. 2B). Pendimethalin and its formulated product had large effects on the transcriptome of MCF-10 A cells by comparison to MCF-7 and MCF-12 A cells (Fig. 3). Transcriptome changes in MCF-7 and MCF-12 A cells were limited as less than 13 genes had their levels altered after exposure to both treatments. In contrast, pendimethalin and its formulation Stomp Aqua altered the expression of 279 and 229 genes in MCF-10 A respectively, indicating a higher treatment sensitivity (Supplementary Data).

The low number of differentially expressed genes in MCF-7 and MCF-12 A cells prevented more in-depth investigation of transcriptome functional changes by pathway enrichment analyses. However, some of the changes were consistent across the cell models and treatments. Three genes were similarly disrupted by pendimethalin and its formulation on MCF-7 (*Rpl21p16* ribosomal protein L21 pseudogene 16, *Vasn* vasorin and *Coro7* coronin 7). Interestingly, *Coro7* also had its expression largely reduced in MCF-12 A cells. As Coro7 is reported to be part of a novel E3 ubiquitin ligase complex, changes in the level of this protein could indicate that pendimethalin effected the ubiquitination machinery.

The large number of genes whose function was altered in MCF-10 A cells by pendimethalin (8 downgulated, 271 upregulated) or Stomp Aqua pendimethalin (11 downgulated, 218 upregulated) treatment, allowed a pathway enrichment analysis to be undertaken. A total of 17 KEGG pathways were enriched and thus characterized the transcriptome alterations caused by pendimethalin, with the top hits being associated with ubiquitin-mediated proteolysis and the function of the spliceosome (Table 1). KEGG pathway enrichment caused by the Stomp Aqua formulation was similar to that seen with pendimethalin alone. This suggests that pendimethalin is responsible for affecting ubiquitin-mediated proteolysis and the function of the spliceosome. These observations further indicate a mechanism by which exposure to this herbicide may lead to breast cancer or promote breast cancer aggressiveness as the expression of spliceosome-related genes are known to be pivotal for breast cancer survival [11]. In addition, dysregulation of ubiquitin-mediated proteolysis can cause alterations in the cell cycle associated with tumour development [12], suggesting yet another mechanism by which pendimethalin could promote breast cancer progression. The results of this study suggesting that pendimethalin can alter ubiquitin-mediated proteolysis, are corroborated by another investigation, which showed that this herbicide has inhibitory effects on proteasome activities at low concentrations [13].

**Table 1:** The most affected KEGG pathways after exposure to pendimethalin or its reference commercial formulation in MCF-10 A predicted from transcriptome analysis. Statistically overrepresented KEGG pathway (adjusted p-value < 0.05) are shown along their adjusted p-value (ns, non significant)

Name	Formulation	Pendimethalin
Ubiquitin mediated proteolysis	0.00009	0.0002
Spliceosome	0.00009	0.0002
Metabolic pathways	0.0001	0.00005
Nucleocytoplasmic transport	0.0001	0.0003
Huntington disease	0.0003	0.002
Cell cycle	0.001	0.0003
Protein processing in endoplasmic reticulum	0.002	0.0008
Parkinson disease	0.005	0.001
Ribosome	0.006	0.0003
Pyrimidine metabolism	0.006	0.008
Endocytosis	0.006	0.03
Lysosome	0.006	0.006
Adherens junction	0.01	ns
Oxidative phosphorylation	0.02	0.006
Wnt signaling pathway	0.03	0.008
mRNA surveillance pathway	0.03	0.001
Inositol phosphate metabolism	0.04	ns
Alzheimer disease	ns	0.01
RNA degradation	ns	0.02
Ribosome biogenesis in eukaryotes	ns	0.02
Valine, leucine and isoleucine degradation	ns	0.03
Renal cell carcinoma	ns	0.04
Nucleotide excision repair (NER)	ns	0.04
Amino sugar and nucleotide sugar metabolism	ns	0.04

The observation that MCF-10 A was more affected comes as somewhat of a surprise because out of the cell lines employed MCF-10 A it is the only one, which does not express estrogen receptor alpha [14, 15]. However, MCF-10 A does express G protein-coupled estrogen receptor 1 (GPER) [16], which has previously been shown to mediate the effect of estradiol on this cell line [17]. Interestingly, this was also the case when the same cells were exposed to bisphenol A and bisphenol A analogue mixtures [8].

In conclusion, this study shows that human mammary epithelial cells, especially non-cancerous MCF-10 A cells, are markedly impacted by exposure to pendimethalin or its commercial formulation Stomp Aqua, with transcriptome analysis suggesting a possible link with breast cancer. Given the lack of information on exposure of human populations to this pesticide, our study prompts the realization of biomonitoring studies to understand if low level exposure to pendimethalin can have endocrine disrupting effects. A deeper understanding of the exposure and mechanisms of action of pendimethalin and Stomp Aqua (and other commercial formulations) is needed.

### Limitations

Endocrine disruptors sometimes do not give a linear dose-response. Our study did not provide information on the concentration relationship of the observed effects. The effects detected in this study are predictions from the classification of the gene function. Additional cellular assays will have to be performed to validate the predictions made from the study of gene expression. Further research studies also need to be conducted in animal models.

## Electronic supplementary material

Below is the link to the electronic supplementary material.

Supplementary Material 1

## Data Availability

All raw data is available in a publicly repository (NCBI GSE182963).
